# DNA replication components as regulators of epigenetic inheritance—lesson from fission yeast centromere

**DOI:** 10.1007/s13238-014-0049-9

**Published:** 2014-04-02

**Authors:** Haijin He, Marlyn Gonzalez, Fan Zhang, Fei Li

**Affiliations:** 1 Department of Biology, New York University, New York, NY 10003 USA; 2 Clinical Translational Research Center, Shanghai Pulmonary Hospital, Tongji University School of Medicine, Shanghai, 200433 China

**Keywords:** epigenetic inheritance, DNA replication, centromere, histone modification, heterochromatin, euchromatin

## Abstract

Genetic information stored in DNA is accurately copied and transferred to subsequent generations through DNA replication. This process is accomplished through the concerted actions of highly conserved DNA replication components. Epigenetic information stored in the form of histone modifications and DNA methylation, constitutes a second layer of regulatory information important for many cellular processes, such as gene expression regulation, chromatin organization, and genome stability. During DNA replication, epigenetic information must also be faithfully transmitted to subsequent generations. How this monumental task is achieved remains poorly understood. In this review, we will discuss recent advances on the role of DNA replication components in the inheritance of epigenetic marks, with a particular focus on epigenetic regulation in fission yeast. Based on these findings, we propose that specific DNA replication components function as key regulators in the replication of epigenetic information across the genome.

## INTRODUCTION

In addition to the genetic code, which is defined by DNA sequence, cells contain another layer of regulatory information, referred to as the “epigenetic code”. The epigenetic code consists mainly of chemical modifications of DNA and histone proteins, and plays an important role in gene expression regulation and genome stability through altering chromatin states (Strahl and Allis, 2000; Couture and Trievel, 2006). Like the genetic code, the epigenetic code can be faithfully transmitted for many generations. This phenomenon is referred to as “epigenetic inheritance” (Zhu and Reinberg, 2011). How genetic information is duplicated and transmitted to subsequent generations is well understood; it is achieved through a series of coordinated interactions between conserved DNA replication components during the S phase of the cell cycle. The process is initiated by the binding of the origin recognition complex (ORC) to replication origins, followed by assembly of a pre-replication complex (preRC), which includes Cdc6, Cdt1, and the MCM complex (Dutta and Bell, 1997; Li and Stillman, 2012). MCM helicase activity is required for unwinding the DNA duplex into single stranded DNA, which leads to the recruitment of proliferating cell nuclear antigen (PCNA) to the replication fork. PCNA functions as a platform for recruitment of a variety of replication factors, including DNA polymerase δ (Pol δ), and Pol ε. Pol ε is important for subsequent leading strand synthesis while Pol δ is required for lagging strand synthesis (Waga and Stillman, 1998; Burgers, 2009). During DNA replication, epigenetic information must also be transmitted to subsequent generations. How epigenetic inheritance is achieved remains poorly understood. In this review, we will discuss recent progress toward our understanding of the role of DNA replication machinery in epigenetic inheritance, particularly on the context of chromatin inheritance at centromeres in the fission yeast *Schyzosaccharomyces pombe*.

**Figure 1:**
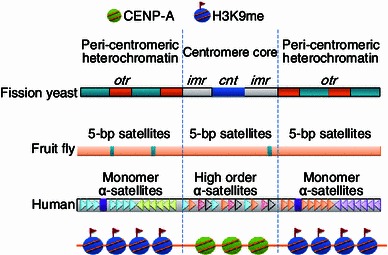
**Schematic of centromere structure in fission yeast,*D. melanogaster*, and humans**. Fission yeast centromere contains a central core region (*cnt*), flanked by *imr* and *otr* repeat regions. Each of the *otr* repeats can be divided into 2 smaller repeat units: *dg* (blue color) and *dh* (orange color). *imr* and *otr* repeat regions are heterochromatic. The sequence and size of DNA repeats in *D. melanogaster* and human centromeres are different from those in fission yeast (Schueler et al., 2001; Sun et al., 2003), but the general chromatin structure and epigenetic profile are conserved among these organisms. At core centromeres, the histone H3 variant, CENP-A, replaces histone H3 in nucleosomes, while histone H3K9 methylation is enriched at the peri-centromeric heterochromatin

Chromatin consists of repeating particles of ~100 Å in size, termed nucleosomes. Nucleosomes are composed of two copies of each of four canonical histone proteins H2A, H2B, H3, and H4. These barrel-like structures wrap around 146 base pairs of DNA, and are linked by histone H1. Strings of nucleosomes fold into higher order chromatin structures that are orderly packaged within the nucleus (Luger, 2003). The centromere is a specific chromatin structure responsible for correct segregation of chromosomes during mitosis and meiosis (Pluta et al., 1995; Amor et al., 2004). In most eukaryotes, centromeres are organized into two distinct domains: a centromeric core, and peri-centromeric heterochromatin that flanks the core (Fig. 1). The centromeric core is where the kinetochore assembles. The kinetochore is a multi-protein complex that mediates the attachment of spindle microtubules to centromeres during chromosomes segregation (McIntosh et al., 2002; Morris and Moazed, 2007; Bloom and Yeh, 2010). Defects in centromere regulation are catastrophic to cells and result in chromosome mis-segregation and an abnormal number of chromosomes, a phenomenon known as aneuploidy (Morris and Moazed, 2007; Weaver and Cleveland, 2007).

Centromeric core chromatin is defined by CENP-A, a centromere-specific histone 3 (H3) variant. CENP-A partially replaces canonical H3 histone at centromeres and is responsible for nucleating the formation of the kinetochore (Palmer et al., 1991; Black and Cleveland, 2011). In most eukaryotic centromeres, the centromeric core contains more than one CENP-A nucleosome, and is therefore bound by multiple spindle microtubules. Such centromeres are referred to as “regional centromeres” (Pluta et al., 1995). In contrast, budding yeast centromeres which consists of a single CENP-A nucleosome bound by a single spindle microtubule, are referred to as “point centromeres”. Point centromeres are defined by the underlying DNA sequence, which consists of 125 bp of DNA wrapped around the sole CENP-A nucleosome (Cottarel et al., 1989). In contrast, in regional centromeres, although their architecture and function are conserved across eukaryotes, their underlying DNA varies significantly in size and sequence across species, suggesting that epigenetic mechanisms are important determinants in their regulation. CENP-A is considered the most likely candidate epigenetic mark for centromere identity and has been the subject of intense study (Henikoff and Furuyama, 2010).

Immediately flanking CENP-A-bound centromeric core is the peri-centromeric heterochromatin. This condensed and transcriptionally silent region is essential for gene regulation, genome stability and chromosome segregation. Peri-centromeric heterochromatin displays a protein binding profile and epigenetic environment that is clearly different from that of centromeric cores. The heterochromatic region is enriched in histone 3 lysine 9 (H3K9) methylation and devoid of H3K4 methylation. In fact, H3K9 methylation is considered an epigenetic hallmark of heterochromatin, conserved from fission yeast to humans (Fig. 1) (Rice and Allis, 2001; Carroll and Straight, 2006). This modification serves as the binding site for the highly conserved chromodomain protein, heterochromatin protein 1 (HP1). HP1 is a classic epigenetic “reader” protein, which can recognize specific epigenetic marks (Rice and Allis, 2001; Taverna et al., 2007). Histones in heterochromatin are also hypo-acetylated. This contrasts with active chromatin (euchromatin) in which H3K4 methylation and hyper-acetylation are enriched (Rice and Allis, 2001).

## CENTROMERES IN FISSION YEAST

Fission yeast has emerged as an excellent model system for cell cycle and chromatin studies in the last several decades. Because of its similarity to mammalian cells at the most fundamental levels, it has gained the nickname of “micro-mammal” (Forsburg and Rhind, 2006). Fission yeast has a relatively small genome of 13.8 Mb harboring ~4800 genes, many of which exist as single copy. Similar to budding yeast, fission yeast is amenable to genetic and biochemical manipulations. Importantly, in contrast to the point centromeres of budding yeast, fission yeast contains “regional centromeres”, which resemble those of higher eukaryotes (Carroll and Straight, 2006). These characteristics make fission yeast especially useful for dissecting the mechanisms governing heterochromatin and centromere structure and function.

Fission yeast contains three chromosomes that range in size from 40 kb to 110 kb. Like in multicellular organisms, centromeres in *S. pombe* consist of a core region (*cnt*, centromere core domain) and peri-centromeric heterochromatin. Centromeric cores span about 4–7 kb and share only limited homology. Immediately flanking the core region are the imperfect repeat regions referred to as innermost repeats (*imr*). *S. pombe*’s CENP-A homolog, Cnp1, is enriched at centromeric cores and *imr* regions. Flanking *imr* regions are the outermost repeat regions (*otr*) also composed of tandem DNA repeats (Chikashige et al., 1989; Allshire and Karpen, 2008). Both *imr* and *otr* regions are heterochromatic in nature. Exogenous marker genes inserted within these regions are silenced, although *otr* regions appear more strongly silenced than *imr* regions (Allshire et al., 1995). Each repeat unit within an *otr* region is composed of the two smaller repeat units, *dg* and *dh* (Wood et al., 2002). Distinct heterochromatin regions are also present in telomeric and mating type loci, which are all rich in tandem DNA repeats.

## REGULATION OF PERI-CENTROMERIC HETEROCHROMATIN

### Role of H3K9 methylation in heterochromatin formation

In fission yeast, like in higher eukaryotes, peri-centromeric heterochromatin is enriched with H3K9 methylation (Fig. 1). This modification is catalyzed by Clr4, a homolog of the mammalian histone methyltransferase, SUV39H1 (Nakayama et al., 2001b). The C-terminal end of Clr4 harbors a SET domain responsible for the enzyme’s catalytic activity, while the N-terminal domain contains a chromodomain region that mediates binding of the enzyme to H3K9 methylation groups (Min et al., 2002; Zhang et al., 2008). Methylating H3K9 is pivotal for heterochromatin formation. Deletion of Clr4 results in loss of H3K9 methylation and heterochromatin silencing. In addition, artificially tethering Clr4 to euchromatic regions is sufficient to induce heterochromatin formation *de novo* (Kagansky et al., 2009).

Previous studies have identified Clr4 as a component of the multi-subunit Clr4 methyltransferase complex (ClrC). In addition to Clr4, the complex also contains Cul4, Rik1, Dos1 (also known Raf1/Cmc1/Clr8), Dos2 (Raf2/Cmc2/Clr7), and Lid2. Disruption of any of these components leads to severe reduction in H3K9me and silencing at heterochromatic regions (Allshire et al., 1995; Hong et al., 2005; Horn et al., 2005; Jia et al., 2005; Li et al., 2005; Thon et al., 2005). Cul4 is a core subunit of Cullin4-based ubiquitin E3 ligase (Jia et al., 2005). Rik1 contains a WD40 domain and is a functional homologue of human DDB1 (UV-damaged DNA binding protein 1) (Horn et al., 2005). Dos1 also contains a WD40 domain. Recent studies showed that Dos1 is a homolog of human DDB1-Cul4 Associated Factor (DCAF) (Buscaino et al., 2012; Kuscu et al., 2014). In humans, DCAF proteins interact directly with DDB1 and function as substrate-recognition receptors for the E3 ligases (Lee and Zhou, 2007). However, the substrate targeted by DDB1-Dos1-Cul4 in fission yeast is still unknown. Lid2 is a conserved H3K4 demethylase important for maintaining H3K4 hypomethylation in heterochromatin. It has been proposed that the ClrC complex may function in coordinating H3K9 methylation and H3K4 demethylation activity in heterochromatin to ensure proper establishment and maintenance of epigenetic states in these regions (Li et al., 2008).

H3K9 methylation in fission yeast is recognized and bound by the human HP1 homologue, Swi6. Like human HP1, Swi6 contains two distinct domains, a chromodomain and a chromoshadow domain. The chromodomain is the “reader” domain that specifically associates with H3K9 methylation, whereas the chromoshadow domain is involved in the dimerization of Swi6 (Cowieson et al., 2000; Bannister et al., 2001). It has been postulated that Swi6 may use a step-wised oligomerization process to generate a high-order multimer that in turn serves as a platform to recruit diverse regulators required for the assembly of chromatin into a less-accessible structure (Fischer et al., 2009). Swi6 also has the ability to recruit cohesin, a protein critical for sister chromatid cohesion (Bernard et al., 2001).

### Histone deacetylation and heterochromatin assembly

Histone deacetylation is another highly conserved feature of heterochromatin in eukaryotes. Fission yeast contains all three subtypes of histone deacetylases (HDACs): Class I (Clr6), Class II (Clr3), and Class III (Sir2), all of which have been shown to be required for heterochromatin silencing. Clr6, a homolog of mammalian HDAC1 and HDAC2 and of Rpd3 in *S. cerevisiae*, deacetylate several lysine residues on histone H3 and H4. Clr6 is essential for viability and regulates heterochromatin silencing and gene activity at euchromatin (Bjerling et al., 2002; Nicolas et al., 2007). Clr3 deacetylates histone H3 on lysine 14, and is a component of the transcriptional silencing complex, SHREC. The SHREC complex physically associates with Swi6, and is important for heterochromatin spreading (Bjerling et al., 2002; Sugiyama et al., 2007). Sir2 is a conserved member of the Sirtuin family of HDACs that uses NAD^+^ as a cofactor. Sir2 in fission yeast can deacetylate multiple lysines in histone H3 and H4, including H3K4, H3K9, H3K14, and H4K16. Recent studies have shown that Sir2 is critical for Clr4-mediated heterochromatin nucleation step and subsequent heterochromatin spreading (Shankaranarayana et al., 2003; Buscaino et al., 2013).

### RNAi and heterochromatin silencing

RNA interference (RNAi), a RNA-induced gene-silencing phenomenon, was originally found to occur at the post-transcriptional level. Core components of the RNAi machinery include Argonaute, Dicer, and a RNA-dependent polymerase. An unexpected yet significant breakthrough was the discovery of a link between RNAi and transcriptional silencing in fission yeast (Volpe et al., 2002). A similar phenomenon was also later discovered in other organisms, including flies (Pal-Bhadra et al., 2004), *Arabidopsis thaliana* (Chan et al., 2004), and *Caenorhabditis elegans* (Guerin et al., 2013). Fission yeast contains a single copy of each RNAi component, namely, Ago1 (Argonaute), Dcr1 (Dicer), and Rdp1 (RNA-dependent polymerase). Remarkably, none of these proteins are essential for viability in fission yeast. However, deletion of any one of these components resulted in loss of H3K9 methylation and silencing at peri-centromeric heterochromatin, indicating the important role of RNAi in heterochromatin silencing (Volpe et al., 2002).

In fission yeast, Ago1, the chromodomain protein, Chp1, and Tas3, which contains a glycine-tryptophan (GW) domain, form the RNA-induced transcriptional silencing (RITS) complex. Like Swi6, Chp1 specifically binds to H3K9 methylation through its chromodomain, thereby anchoring the RITS complex to heterochromatin (Motamedi et al., 2004). Consistent with this, the level of siRNAs is reduced in mutants of the ClrC complex, presumably due to dissociation of the RITS from H3K9me-deficient heterochromatin in these mutants (Motamedi et al., 2004; Hong et al., 2005; Li et al., 2005). Tas3 serves as a backbone for the RITS complex, linking Chp1 and Ago1 (Schalch et al., 2011). The C-terminal end of Tas3 contains an α-helical motif which can assemble into a helical polymer that is essential for the *cis* spreading of the RITS complex at peri-centromeres (Li et al., 2009). Rdp1 associates with Cid12, a putative polyA polymerase, and the Hrr1 helicase, to form the RNA-directed RNA polymerase (RDRC) complex (Motamedi et al., 2004). Heterochromatin transcripts are processed into small single stranded RNA fragments through the slicer activity of Ago1 in the RITS complex (Irvine et al., 2006). These fragments are then used by RDRC to prime the synthesis of double stranded long non-coding RNAs, which are subsequently processed into siRNAs by Dicer (Motamedi et al., 2004). SiRNAs have been proposed to act as key nucleation factors for heterochromatin assembly by recruiting the ClrC complex to heterochromatin. Stc1, a LIM domain protein, interacts with both the ClrC complex and RNAi components to couple RNAi to H3K9 methylation at peri-centromeres (Bayne et al., 2010).

### Cell cycle and RNAi

Previous studies have shown that phosphorylation of histone H3 at serine 10 (H3S10P) antagonizes the binding of HP1 to H3K9me, and that the binary phospho-methyl switch between the two modifications is crucial for heterochromatin assembly in humans (Fischle et al., 2003). In fission yeast, H3S10 phosphorylation increases during mitosis and results in dissociation of Swi6 from H3K9me sites during early S phase. At the same time, H3K9me is maximally reduced and H3K4me, the epigenetic mark for active transcription, increases at this stage. Correspondingly, heterochromatin transcription becomes active during early S phase. This coincides with the timing of replication of centromeres, which in *S. pombe* differs from the timing of replication of the other major constitutive heterochromatin domains, namely telomeres and mating type loci (Chen et al., 2008; Kloc et al., 2008). Heterochromatin in fission yeast is transcribed by RNA Pol II. Consistent with this, Pol II was found to be recruited to centromeric repeats during early S phase. Transcripts from both strands of *dg* and *dh* regions are immediately processed into small RNAs by RNAi machinery. Following the increase of siRNAs during early S phase, H3K9me begins to increase at late S phase, and reaches the highest level at G_2_ phase (Chen et al., 2008; Kloc et al., 2008). During S phase, key ClrC components, including Rik1 and Dos2 are also recruited to peri-centromeric heterochromatin (Chen et al., 2008). These findings revealed that heterochromatin assembly is dynamic throughout the cell cycle, and pointed to the S phase of the cell cycle as a crucial stage for establishment and propagation of heterochromatic epigenetic marks.

## DNA REPLICATION AND INHERITANCE OF HETEROCHROMATIN SILENCING

### A role for the DNA Pol ε subunit, Cdc20, in inheritance of H3K9 methylation

Co-regulation of RNAi and the S phase of the cell cycle is intriguing. Recent studies provide clues into how RNAi and heterochromatin formation are coupled with DNA replication, thus providing mechanistic insight into how H3K9 methylation is inherited. Using mass spectrometry analysis, Li et al., demonstrated that the Dos2 and Rik1 components of the ClrC complex associate with the DNA replication factor, Cdc20, and the HEAT domain containing protein, Mms19 (Li et al., 2011a). Cdc20 is the catalytic subunit of DNA Pol ε (Durso and Nurse, 1997), and Mms19 is involved in diverse functions, including DNA repair, transcription, chromosome segregation, and the Fe-S pathway (Wu et al., 2001; Hatfield et al., 2006; Ito et al., 2010; Papatriantafyllou, 2012). Li et al. further revealed that the complex is crucial for assembly of heterochromatin during S phase. Disruption of Cdc20 results in severe loss of H3K9me and heterochromatin silencing. Specific association of Dos2 and Rik1 with peri-centromeric heterochromatin is also lost in a *cdc20* mutant, suggesting that Cdc20 may play an essential role in recruiting the ClrC complex to heterochromatin. Furthermore, the Dos2-Rik1-Cdc20 complex is important for the regulation of transcription of heterochromatic siRNAs during S phase (Li et al., 2011a). These findings demonstrated a key role for Cdc20 in RNAi-dependent heterochromatin assembly during S phase, and shed light onto how DNA replication, RNAi and the ClrC complex may be coordinated to process H3K9me and heterochromatin inheritance during DNA replication. Another study shows that during S phase of the cell cycle, RNAi-dependent processing of nascent heterochromatin transcripts promotes degradation of these transcripts and releases RNAPII from heterochromatin (Zaratiegui et al., 2011). This is important because it prevents potential collisions between transcription and DNA replication machinery, and allows the replication fork to continue advancing concomitantly with the recruitment of the ClrC complex by the DNA polymerase subunit, Cdc20.

### Initiation of heterochromatin formation: RNAi or Cdc20-directed?

Small RNAs generated via the RNAi pathway have been proposed to serve as nucleating factors for heterochromatin assembly and to recruit the H3K9-methylating complex, ClrC (Verdel et al., 2004). However, H3K9 methylation is not completely abrogated in RNAi mutants and recruitment of the H3K9me-binding protein, Swi6, to heterochromatin is only weakly affected (Hall et al., 2003; Li et al., 2005). In contrast, H3K9me is completely lost, and Swi6 dissociates from heterochromatin in cells lacking components of the ClrC complex. In addition, in cells in which both Clr4 and the RNAi pathway are disrupted, overexpression of Clr4 bypasses the RNAi defect and results in the deposition of H3K9me at peri-centromeric heterochromatin (Shanker et al., 2010). In support of this, artificial tethering of Clr4 to a euchromatic locus was found to be enough to induce heterochromatin assembly (Kagansky et al., 2009), further confirming that H3K9me can occur independently of RNAi. These findings suggest the existence of a pathway for heterochromatin inheritance that acts independently of RNAi. In this regard, initiation of heterochromatin assembly by the ClrC complex has been proposed as an alternative model. In support of this view, components of the ClrC complex, such as Rik1, have been shown to be absolutely essential for H3K9 methylation and able to bind chromatin independently of RNAi to nucleate heterochromatin assembly (Reyes-Turcu et al., 2011).

However, how the ClrC complex is recruited to peri-centromeric heterochromatin in the first place remains unresolved. Here we propose that Cdc20, the DNA Pol ε subunit, as a central player driving the initiation of heterochromatin assembly. During DNA replication, Cdc20 recruits the ClrC complex to nascent heterochromatin resulting in the deposition of H3K9me at nucleation sites. Small RNAs generated from peri-centromeric transcripts may further facilitate and reinforce Cdc20-directed targeting of the ClrC complex to nascent heterochromatin during S phase by base pairing with heterochromatic DNA.

### Other replication components implicated in heterochromatin silencing

In addition to Cdc20, other DNA replication factors in fission yeast implicated in heterochromatin silencing include, Orc and DNA Pol α subunits, Swi7, and Mcl1. Perturbing these factors results in loss of silencing at peri-centromeric heterochromatin. All of these factors were also found to interact with the homolog of human HP1 in fission yeast, Swi6 (Nakayama et al., 2001a; Natsume et al., 2008; Li et al., 2011b). Cdc18, the homolog of human Cdc6, also interacts with Swi6. Cdc18 is required for the association of Swi6 with peri-centromeric heterochromatin but appears to be dispensable for heterochromatin silencing (Li et al., 2011b). The exact role of these replication factors in heterochromatin formation and inheritance of H3K9 methylation remains unclear.

## DNA REPLICATION AND INHERITANCE OF CENP-A

Numerous studies have shown that CENP-A is essential for centromere structure and function, and acts as the epigenetic mark for centromeres. Unlike canonical histones, which are deposited into chromatin during S phase of the cell cycle, parental and newly synthesized CENP-As are assembled into core centromeres at different stages of the cell cycle: parental CENP-A is partitioned equally and incorporates into daughter centromeres following DNA replication during S phase, whereas newly synthesized CENP-A is loaded at centromeres only during later stages of the cell cycle (Jansen et al., 2007; Schuh et al., 2007; Black and Cleveland, 2011; Bui et al., 2012; Shivaraju et al., 2012). Assembly of newly synthesized CENP-A chromatin requires the histone chaperone, HJURP/Scm3 (Camahort et al., 2007; Foltz et al., 2009). Little is known about how the parental CENP-A is faithfully recruited to centromeric nucleosomes during DNA replication. In fission yeast, CENP-A loading during S phase requires Ams2, a cell-cycle-regulated GATA-type factor (Chen et al., 2003). A recent study demonstrated that Cdc20 interacts with Ams2 through Dos1/2 to mediate CENP-A incorporation during S phase. Disruption of the Cdc20-Dos1/2 complex leads to dissociation of CENP-A from centromeres and severe chromosome segregation defects (Gonzalez et al., 2013). This study suggests that epigenetic inheritance of core centromeres also depends on DNA replication components and provides mechanistic insight into how the parental CENP-A is inherited during S phase.

## DNA REPLICATION FACTORS AS PART OF EPIGENETIC REPLICATION MACHINERY

Studies from various organisms implicate DNA replication factors in the inheritance of heterochromatin silencing. For instance, in budding yeast, mutations in components of the ORC complex affect transcriptional silencing (Foss et al., 1993; Bell et al., 1995; Fox et al., 1995). In *Drosophila* and humans, ORC2 interacts with HP1, and depletion of ORC2 in humans results in dissociation of HP1 from heterochromatin (Pak et al., 1997; Shareef et al., 2001; Prasanth et al., 2004; Prasanth et al., 2010). In budding yeast, MCM10 was found to interact with Sir2 and mutations in MCM proteins result in a reduction in heterochromatin silencing (Dziak et al., 2003; Liachko and Tye, 2005). In *Drosophila*, Mcm10 is known to bind HP1 (Christensen and Tye, 2003). In addition, disruption of PCNA in both budding yeast and *Drosophila* causes heterochromatin defects (Henderson et al., 1994; Zhang et al., 2000). In mammals, PCNA interacts with chromatin modification enzymes, including DNMT1, a DNA methyltransferase, and SET8/Pr-Set7, a histone methyltransferase for H4K20me, both of which are important for heterochromatin assembly (Chuang et al., 1997; Huen et al., 2008). Notably, DNA polymerases, including Pol α and Pol ε, have also been demonstrated to be involved in heterochromatin silencing in budding yeast and *A. thaliana* (Ehrenhofer-Murray et al., 1999; Yin et al., 2009; Liu et al., 2010; Hyun et al., 2013). In addition to the effect on heterochromatin and centromeres, DNA replication machinery has also been implicated in the epigenetic regulation of open chromatin: in human cells, PCNA was found to target ISWI, a chromatin-remodeling complex, to open chromatin during replication to mediate maintenance of active chromatin states (Poot et al., 2004).

**Figure 2:**
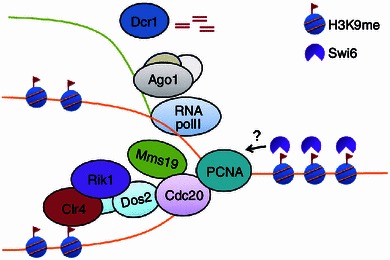
**Model of inheritance of H3K9 methylation during DNA replication**. During S phase of the cell cycle, Cdc20, the catalytic subunit of Pol ε, travels with the replication fork to synthesize the leading strand DNA. When it reaches heterochromatin, Cdc20 may sense the epigenetic state of parental nucleosomes by interacting, directly or indirectly, with the epigenetic reader protein, Swi6/HP1, associated with parental histones. This leads to recruitment of the ClrC complex to replication forks. Histone modification enzymes in the ClrC complex, including Clr4 and Lid2, modify the histones on daughter DNA to ensure hypermethylation of H3K9. Meanwhile, Cdc20 recruits the transcription regulator, Mms19, to heterochromatin, which in turn promotes heterochromatin transcription by RNA Pol II. These transcripts are processed by RNAi machinery into siRNAs, which facilitate the formation of heterochromatin after replication

Based on these observations, we propose that specific DNA replication components act as central regulators in the inheritance of epigenetic information throughout the genome. We envision such components acting in a coordinated fashion during S phase to recruit the appropriate histone modification or DNA methylation enzymes, required for the faithful inheritance of epigenetic marks. The advantage of sharing the same machinery for the replication of both genetic and epigenetic information is obvious since it allows effective coupling of DNA replication and epigenetic inheritance. However, given that epigenetic patterns vary throughout different regions of the genome, how can general DNA replication factors command the inheritance of diverse epigenetic states? One possibility is that DNA replication components are able to “sense” parental epigenetic states by interacting with specific epigenetic “reader” proteins. This, in turn, leads to recruitment of proper “writer” proteins that modify daughter DNA or histones (Fig. 2). Consistent with this idea, multiple replication components have been shown to be associated with the classic reader protein, Swi6/HP1 (Nakayama et al., 2001a; Christensen and Tye, 2003; Prasanth et al., 2010).

## CONCLUDING REMARKS

One fundamental question in the field of epigenetics is how epigenetic states are faithfully transmitted through generations. Recent studies in fission yeast and other organisms provide significant insight into the role of DNA replication machinery in epigenetic inheritance. In the future, it is important to elucidate the mechanism for how different DNA replication components coordinate to mediate chromatin inheritance. Another important question is how DNA replication machinery guides the inheritance of various chromatin states. Furthermore, DNA replication is also known to provide a critical window of opportunity for changing epigenetic states, which can drive cellular differentiation during development (Probst et al., 2009). The molecular mechanism by which this epigenetic reprogramming takes place remains obscure. Future experiments are needed to further address these important questions.

## Abbreviations

ClrC: Clr4 methyltransferase complex
HDACs: histone deacetylases
HP1: heterochromatin protein 1
H3K9: histone 3 lysine 9
ORC: origin recognition complex
PCNA: proliferating cell nuclear antigen
Pol δ: polymerase δ
Pol ε: polymerase ε
RNAi: RNA interference
